# 

**Published:** 1914-01-03

**Authors:** 


### CONTENTS.

PAGE

**The Voluntary Hospitals' Outlook** ... ... 355

**Board of Education and the Tuberculous Child** 356

**Hospital and Institutional News** ... ... ... 357

State Hospitals Won't be Tolerated by Englishmen—A Central Hospital Board for Bradford?—Another Fortune for the Hospitals—Mount Vernon Hospital Buildings—The Future of Banstead Mental Hospital—Mumps in the City—Possibility of Rabies in England—The Sale of Cocaine Snuff—Santa Clause in a Monoplane—The Late Sir Trevor Lawrence—Modern Absurdities—The British Hospitals Association—The Growth of Medical Trade Unionism—Sir John Tuke's Bequests—Infirmaries and the Insurance Act—Water Analysis on the Cheap—Patients and their Beauty Sleep—Fifty-two Years at Guy's Hospital—Endowment of Beds by Instalments—Latest Report of Addenbrooke's Hospital—A Hospital History Continued—Dr. Mackintosh's "Coming of Age"—Truthfulness without Tact—The Dust Fall of London—A Grave Scandal.

This Week's Drug Market.

PAGE

**A New Medico-Psychological Institution** ... 363

By EDWIN L. ASH, M.D.

**The Control of the Invalid Children's Aid Association** ... ... ... ... ... ... 364

**The Year's Work: "The Hospital's" Review of 1913** ... ... ... ... ... ... 365

**A Medical Retrospect of 1913** ... ... ... 367

The Lessons of Experience.

**Report on the Norfolk and Norwich Hospital** (illustrated) ... ... ... ... ... ... 369

By Sir HENRY BURDETT, K.C.B., K.C.V.O.

**Buildings Contemplated and in Progress** ... 372

**The Scheme for Raising Funds at Beckett Hospital, Barnsley** ... ... ... ... 378

[*See over.*]

### CONTENTS—(*continued*).

PAGE

**Christmas in the Hospitals** ... ... ... ... ... 373

- Punch and Judy in the City Road.

- Tango Decorations at Leeds.

- My First Christmas in Hospital. By a Patient aged Ten Years.

- Santa Claus in Great Ormond Street.

- A Ward Snowstorm at Bath.

- "Peter Pan" in a Private Mental Hospital.

- The First Christmas at Dagenham Sanatorium.

- "A Christmas Dream" at Birmingham.

- Novel Decorations at Wakefield Mental Hospital.

- An Epidemic and a Play at Manchester.

- Without Decorations at Grimsby.

- Children's Day at the Metropolitan.

- Another Sanatorium Christmas.

**The Editor's Letter-Box** ... ... ... ... ... 379

- What will the Profession Gain by Combinations?

**Institutional Needs** ... ... ... ... ... 379

PAGE

**The Profession of Medicine** ... ... ... 380

- The National Medical Union.

**Appointments** ... ... ... ... ... ... 382

**The Ansell Fortune** ... ... ... ... ... 382

**The Institutional Worker** ... ... (Suppl.) 1
- Some Hints upon the Annual Report.

**Our Bureau of Information** ... ... ,, 2

- Questions for January.

- Employment and Training.

- The Sick and in Need.

- Practical Matters.

**Approved Homes** ... ... ... ,, 3

**Gifts and Bequests** ... ... ... ,, 3

**The Book Market** ... ... .. ,, 3

